# Cannabinoids Regulate Sensory Processing in Early Olfactory and Visual Neural Circuits

**DOI:** 10.3389/fncir.2021.662349

**Published:** 2021-07-07

**Authors:** Thomas Heinbockel, Alex Straiker

**Affiliations:** ^1^Department of Anatomy, Howard University College of Medicine, Washington, DC, United States; ^2^The Gill Center for Biomolecular Science and the Department of Psychological and Brain Sciences, Indiana University, Bloomington, IN, United States

**Keywords:** depolarization-induced suppression of inhibition, marijuana, retina, synaptic plasticity, dendrodendritic, odor, neuromodulation, smell

## Abstract

Our sensory systems such as the olfactory and visual systems are the target of neuromodulatory regulation. This neuromodulation starts at the level of sensory receptors and extends into cortical processing. A relatively new group of neuromodulators includes cannabinoids. These form a group of chemical substances that are found in the cannabis plant. Δ9-tetrahydrocannabinol (THC) and cannabidiol (CBD) are the main cannabinoids. THC acts in the brain and nervous system like the chemical substances that our body produces, the endogenous cannabinoids or endocannabinoids, also nicknamed the brain’s own cannabis. While the function of the endocannabinoid system is understood fairly well in limbic structures such as the hippocampus and the amygdala, this signaling system is less well understood in the olfactory pathway and the visual system. Here, we describe and compare endocannabinoids as signaling molecules in the early processing centers of the olfactory and visual system, the olfactory bulb, and the retina, and the relevance of the endocannabinoid system for synaptic plasticity.

## Introduction

Along their pathways, neural elements of sensory systems are targeted by a variety of modulatory regulators. A relatively new group of neuromodulators includes cannabinoids. These form a group of chemical substances that are found in the cannabis plant. Δ9-tetrahydrocannabinol (THC) and cannabidiol (CBD) are the main cannabinoids. THC acts in the brain and nervous system like the chemical substances that our body produces, the endogenous cannabinoids or endocannabinoids (eCBs), also nicknamed the brain’s own cannabis (Nicoll and Alger, 2004). The two cannabinoid receptors, CB_1_ and CB_2_, together with the eCBs, form the endocannabinoid system (Iannotti et al., 2016). The eCB system was first discovered because it can be activated by a plant-derived compound, namely, THC, the bioactive ingredient of cannabis (Ameri, 1999). Although cannabinoid receptors can be artificially activated by THC, CB_1_, exist in all normal brains (Herkenham et al., 1991; Matsuda et al., 1993) and subserve many essential brain functions when activated by their natural ligands, eCBs, e.g., motor behavior, learning, memory, cognition and pain reception. The endocannabinoid system has emerged as a critical regulatory system for many bodily functions in health and disease (Di Marzo and Petrosino, 2007). Furthermore, endocannabinoids are increasingly considered as neuroprotective agents (Lafreniere and Kelly, 2018; Baul et al., 2019; Gonçalves et al., 2020; Junior et al., 2020). The study of the endocannabinoid system has the potential to pave the way for developing cannabis-related substances as medications and cannabinoid-based therapies in the treatment of various brain disorders.

Endocannabinoids are derived from membrane lipids and activate cannabinoid receptors. The two main eCBs that have been primarily implicated in cannabinoid signaling are 2-arachidonoyl glycerol (2-AG, Mechoulam et al., 1995; Sugiura et al., 1995) and arachidonoyl ethanolamine (AEA, anandamide, Devane et al., 1992). These lipid messengers are derived from membrane lipids upon neuronal activation and are broken down enzymatically, extracellularly after receptor activation. Endocannabinoids are part of a larger family of lipids that have been hypothesized to play physiological roles in the body (Piomelli, 2003).

Many CB_1_ expressing neurons in the CNS are GABAergic (Tsou et al., 1998). In these cases, eCBs activate CB_1_ at presynaptic terminals to reduce transmitter release, either glutamate (Lévénés et al., 1998; Takahashi and Linden, 2000; Kreitzer and Regehr, 2001b) or GABA (Katona et al., 1999; Hoffman and Lupica, 2000; Ohno-Shosaku et al., 2001; Varma et al., 2001; Wilson and Nicoll, 2001; Diana et al., 2002). Endocannabinoids mediate a type of short–term synaptic plasticity, originally observed in the hippocampus and cerebellum (Kreitzer and Regehr, 2001a; Wilson and Nicoll, 2001), namely DSI (Depolarization-induced Suppression of Inhibition). In DSI, depolarized principal neurons release eCBs that travel to presynaptic inhibitory interneurons, activate CB_1_ at presynaptic terminals, and subsequently, transiently reduce presynaptic firing and neurotransmitter (GABA or glutamate) release. A similar CB_1_-mediated phenomenon, Depolarization-induced Suppression of Excitation (DSE) was observed at excitatory synapses onto Purkinje cell synapses in the cerebellum (Kreitzer and Regehr, 2001b). The retrograde signaling of endocannabinoids has become a hallmark feature of this signaling system.

This review is structured in the following manner: first, the circuitry of two sensory systems, olfactory and visual, will be outlined, followed by a description of the components of the endocannabinoid system within the circuitry. The eCB-mediated neuromodulation of that circuitry and behavioral effects will be covered next. The review will conclude with a comparison of key findings for both sensory systems and an outlook for future research questions.

## Comparison of Early Olfactory and Visual Processing

**Figure 1:**
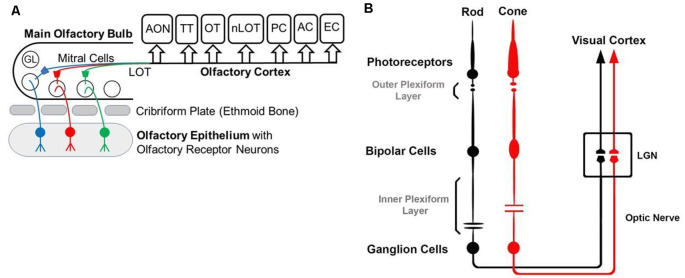
Diagrams of the olfactory **(A)** and visual **(B)** pathway. **(A)** GL—glomerulus, LOT—lateral olfactory tract, AON—anterior olfactory nucleus, OT—olfactory tubercle, PC—piriform cortex, EC—entorhinal cortex, AC—amygdaloid complex, TT—tenia tecta, nLOT—nucleus of Lateral Olfactory Tract. **(B)** Simplified visual pathway of light responses through photoreceptors, bipolar cells, and ganglion cells and on to lateral geniculate nucleus (LGN) in the thalamus. Retinal horizontal and amacrine cells are omitted to highlight the pathway of the light response.

**Figure 2:**
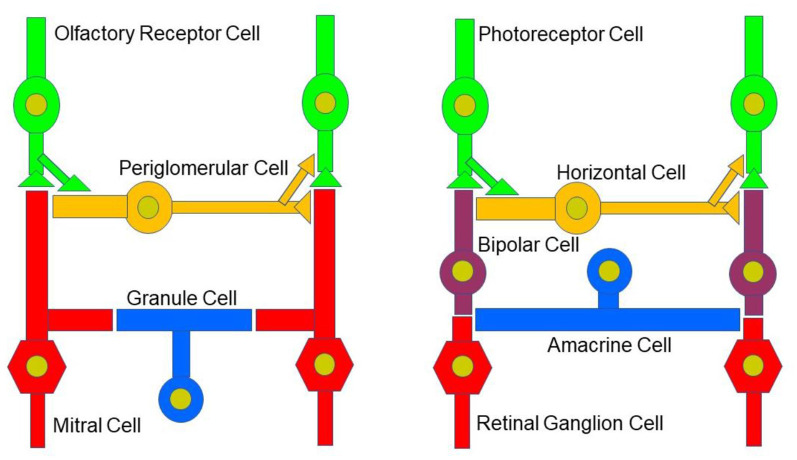
Neural circuitry in the early olfactory and visual system. Early sensory processing engages similar circuit mechanisms in both systems and can be depicted in a similar manner.

While the basic signaling functions of the endocannabinoid system are understood relatively well in limbic structures such as the hippocampus and the amygdala, this signaling system is less well understood in the olfactory pathway (Terral et al., 2020; Heinbockel et al., 2021) and visual system. Here, we describe the early olfactory and visual pathways and compare and contrast endocannabinoids as signaling molecules in them and the relevance of the endocannabinoid system for synaptic plasticity. In the olfactory system, the focus is on the glomerular layer of the main olfactory bulb, the first central relay station for olfactory information coming from olfactory receptor cells in the nose. In the visual system, the focus is on retinal circuits (Figure 1). Even though the olfactory system is distinctly different from the visual system in structure and function, early sensory processing engages similar circuit mechanisms in both systems, such as lateral inhibition, feedforward, and feedback inhibition and excitation, and convergence of sensory neurons on downstream output neurons (Figure 2). The two sensory systems utilize similar computational transformations, suggesting that the main olfactory bulb is directly comparable to the retina, albeit the underlying algorithms mediating the transformations are different because of different physical properties of the stimuli of the two systems (Cleland, 2010). A simplified circuit diagram of the neural elements of this early processing looks very similar in both systems (Figure 2). A comparison of these two sensory systems at their first synaptic relays and its neural circuitry appears to be rewarding with regard to eCB modulation.

## Neural Circuitry in The Main Olfactory System

The olfactory pathway starts in the deep in the nasal cavity where our organ of smell is formed as a specialized epithelium, the olfactory epithelium which sits on the superior conchae and presents as the olfactory area. Each nasal cavity has its own olfactory area in the roof of the nose. The olfactory epithelium is a pseudostratified ciliated columnar epithelium and houses olfactory receptor neurons, supporting cells (sustentacular cells), and basal stem cells. Olfactory receptor cells are bipolar neurons with cilia emanating from their dendrite. Odorant receptor proteins in the membranes of the cilia bind and detect odorant molecules (Bushdid et al., 2014). The axons of olfactory receptor cells form the olfactory nerve, cranial nerve I, that traverses the cribriform plate of the ethmoid bone, and projects to the ipsilateral olfactory bulb. There, the axons synapse on central neurons in olfactory glomeruli, the input centers in the main olfactory bulb. Odorant molecules that are inhaled when we breathe, bind to odorant receptor proteins, thereby transducing odorant molecules into intracellular signals which activate olfactory receptor neurons. Odorant receptor proteins form a large gene family of G-protein coupled receptors that are expressed in the olfactory epithelium (Buck and Axel, 1991; Young et al., 2002; Buck, 2005; Axel, 2005). There are more than 1,000 genes in the mammalian genome that encode the many different odorant receptor proteins. However, not all of them are expressed and functional. In mice, more than 1,400 genes including about 300 pseudogenes are found in this odorant receptor multigene family, whereas the gene family consists of around 400 functional and 600 pseudogenes in humans (Gilad and Lancet, 2003; Niimura, 2009; Mainland et al., 2013; Hayden and Teeling, 2014; Barnes et al., 2020). Despite the large number of olfactory receptor genes in the genome, a given olfactory receptor cell expresses only one of them (Buck and Axel, 1991). Moreover, in mice, the expression pattern of olfactory receptor genes presents itself as four different zones of the olfactory epithelium (Buck, 1993; Ressler et al., 1993; Sullivan et al., 1994) such that olfactory receptor cells that express the same olfactory receptor are found in only one of the four zones. The olfactory epithelium houses several million olfactory receptor cells. In mice, the ones that express the same olfactory receptor project their axon to the same one or two glomeruli in the olfactory bulb, where the axon terminals form synaptic contacts onto central neurons.

Olfactory receptor nerve terminals synapse on principal neurons of the main olfactory bulb, mitral and tufted cells, as well as juxtaglomerular cells in olfactory glomeruli. In the mouse, about 2000 glomeruli are present in each of the two olfactory bulbs. The glomeruli in the olfactory bulbs have been hypothesized to be organized chemotopically (Sharp et al., 1975; Friedrich and Korsching, 1997), such that a glomerulus could be a discrete functional unit and serves as an anatomical address to collect and process specific molecular features about the olfactory environment, conveyed to it by olfactory receptor cell axons expressing specific olfactory receptor proteins (Buonviso and Chaput, 1990; Buck, 1996; Mombaerts, 1996). However, this concept has been challenged (Ma et al., 2012) such that the olfactory bulb representation of chemical features is spatially distributed without chemotopy. In addition, these authors found no correlation between odor-evoked-pattern of activity and odor structure. Instead, they observed that structurally related odors can be represented by ensembles of spatially distributed glomeruli. Since glomeruli are tuned to odors from multiple classes, Ma et al. suggest that glomeruli are hierarchically arranged into clusters according to their odor-tuning similarity (Ma et al., 2012). Each glomerulus has a shell of interneurons and glial cells (McQuiston and Katz, 2001), inside of which the dendrites of interneurons and output neurons receive olfactory receptor cell input (Pinching and Powell, 1971a, b; White, 1972, 1973).

The glomerular interneurons are collectively called juxtaglomerular cells and comprise several types of neurons that send dendrites into the glomerular neuropil (Pinching and Powell, 1971a,b,c; Shipley and Ennis, 1996; Ennis et al., 2007). These cell types include external tufted cells, “short axon” cells, and periglomerular cells. Periglomerular cells and short-axon cells are considered interneurons, even though short-axon cells form extensive interglomerular connections (Kiyokage et al., 2010). Periglomerular cells are GABAergic interneurons and form a heterogeneous neuron population with different firing patterns and morphological properties (Shao et al., 2009; Kiyokage et al., 2010). Short axon cells express both GABA and dopamine, and external tufted cells are glutamatergic (Ribak et al., 1977; Hayar et al., 2004; Kiyokage et al., 2010). Olfactory receptor cell axons also synapse on output neurons, the mitral/tufted cells. In olfactory bulb glomeruli, estimates range from about 10 to 40 mitral cells that innervate each glomerulus and project their axon out of the olfactory bulb (Dhawale et al., 2010; Sosulski et al., 2011; Ke et al., 2013). Mitral cells that innervate a specific glomerulus typically respond to a specific set of odorants. Odorant identity is determined by the olfactory receptor cells that are activated in the olfactory epithelium in response to odor stimulation. An odor is encoded through the combination of activated olfactory receptor cells, where each olfactory receptor detects a molecular feature of the odorant.

Deeper to the glomerular layer, the main olfactory bulb includes, in sequence, the external plexiform layer, the mitral cell layer, the internal plexiform layer, and the granule cell layer. Dendrodendritic synapses are a prominent circuit feature in the main olfactory bulb. One example is periglomerular cells that receive glutamatergic input from the olfactory nerve or dendrodendritic glutamatergic input from external tufted or mitral cells (Pinching and Powell, 1971b; Shipley and Ennis, 1996; Hayar et al., 2004; Ennis et al., 2007). In turn, periglomerular cells presynaptically inhibit olfactory receptor neurons (Aroniadou-Anderjaska et al., 2000; Murphy et al., 2005) and postsynaptically regulate mitral and tufted cell activity (Dong et al., 2007) through GABAergic transmission. Mitral and tufted cells form output channels from the main olfactory bulb to the olfactory cortex. The term olfactory cortex refers to those areas in the rostro-ventral portion of the forebrain that receive direct projections from the main olfactory bulb (Fontanini, 2009; Wilson and Rennaker, 2010). This includes the anterior olfactory nucleus (also referred to as the anterior olfactory cortex), the olfactory tubercle, the cortical nucleus of the amygdala, the piriform cortex, the tenia tecta, the nucleus of the lateral olfactory tract, and lateral regions of the entorhinal cortex that receive minor direct input from the main olfactory bulb (Neville and Haberly, 2004; Wilson and Rennaker, 2010). The piriform cortex is not only the largest cortical area that is primarily involved in the perception and learning of olfactory stimuli, it is also the most important higher-order brain center for olfactory processing and receives most of the main olfactory bulb projections. The piriform cortex has an evolutionarily well-conserved cellular and synaptic organization and is considered as a paleocortex because of its old phylogeny (Haberly, 1990). Not only does the olfactory cortex receive input from the main olfactory bulb, but it also forms reciprocal relationships with limbic areas, such as the amygdala (Majak et al., 2004), the hypothalamus (Price et al., 1991), and the perirhinal cortex (Luskin and Price, 1983; reviewed in Wilson and Rennaker, 2010).

While the main olfactory bulb sends axons to higher-order olfactory centers (afferent fibers), even more, centrifugal axons, originating in higher brain centers, innervate different cell layers in the main olfactory bulb (efferent fibers; Swanson, 2004; Kiselycznyk et al., 2006; Laaris et al., 2007). These centrifugal neurons provide olfactory and/or modulatory feedback to neurons in the main olfactory bulb which is important for experience-dependent modulation (Kiselycznyk et al., 2006). Centrifugal projections include glutamatergic projections from the olfactory cortical (anterior piriform cortex, anterior olfactory nucleus), frontal cortex, and hippocampal structures (deOlmos et al., 1978; Davis and Macrides, 1981; Luskin and Price, 1983). These bulbo-cortical loops are thought to be important for maintaining the oscillatory dynamics of the main olfactory bulb (Gray and Skinner, 1988; Neville and Haberly, 2003; Martin et al., 2006). The modulatory centrifugal neurons originate in the locus coeruleus (noradrenergic—norepinephrine), the horizontal limb of the diagonal band of Broca (cholinergic—acetylcholine, GABAergic—GABA), and the raphe nucleus (serotonergic—serotonin) (Macrides et al., 1981; Halasz, 1990; Shipley et al., 1995; Shipley and Ennis, 1996; Cleland and Linster, 2003). Cortical responses to odor are shaped by the limbic and modulatory connections along the olfactory pathway. Olfactory information from the main olfactory bulb is transformed in cortical circuits which depends on an associative network originating in the piriform cortex (Pashkovski et al., 2020).

## CB_1_ Expression and Cannabinoid Enzymatic Machinery in Olfactory Circuits

**Figure 3:**
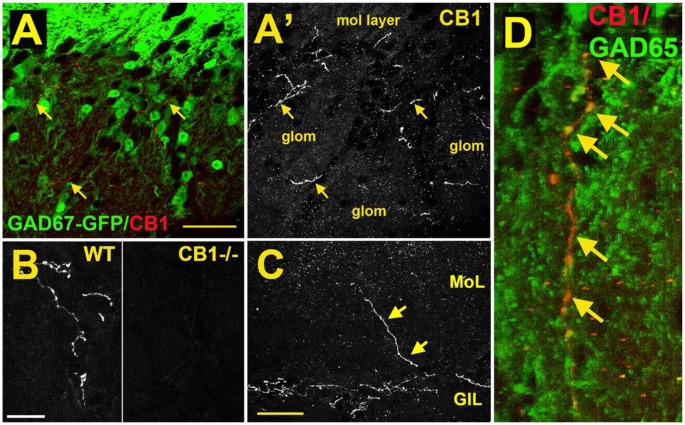
CB_1_ receptors are present in a sub-population of GAD65-positive periglomerular neurons of murine main olfactory bulb. **(A)** Micrograph shows GAD67-GFP (green) and CB_1_staining (red, arrows) in the glomerular layer of the main olfactory bulb. CB_1_ and GAD67-GFP staining does not overlap. **(A’)** CB_1_ staining from **(A)** shows that the staining for CB_1_ is restricted to a few neuronal processes. EPL—external plexiform layer, glom—glomerulus. **(B)** CB_1_ staining in sample WT and CB_1_^−/−^ tissue taken at the same setting. **(C)** Micrograph shows rare process extending to the external plexiform layer. **(D)** Projection of a Z series of GAD65 (green) and CB_1_ (red) staining shows a long overlapping process (overlap in yellow, arrows). Scale bars: **(A)**: 30 μm; **(B)**: 20 μm; **(C)**: 35 μm; **(D)**: 10 μm. Adapted from Wang et al. (2019).

As a first step to determine the potential role of the cannabinoid system in the main olfactory bulb, the receptor expression of CB_1_ was assessed with the use of an antibody against the CB_1_ receptor (Soria-Gómez et al., 2014; Freundt-Revilla et al., 2017; Wang et al., 2019). Cells in the glomerular layer of the main olfactory bulb were shown to express NAPE-PLD, an enzyme implicated in the synthesis of anandamide (Okamoto et al., 2007; Egertová et al., 2008). However, the Allen Brain Atlas revealed little message for the 2-AG-synthesizing enzymes diacylglycerol lipase alpha (DAGLα) or beta (DAGLβ; Allen Institute for Brain Science, 2009). Other works using immunohistochemistry and autoradiography indicate that CB_1_ is present in the main olfactory bulb with moderate to intense levels of staining (Herkenham et al., 1991; Pettit et al., 1998; Tsou et al., 1998; Moldrich and Wenger, 2000; Freundt-Revilla et al., 2017). Moldrich and Wenger (2000) described a moderate density of CB1 immunoreactive cell bodies and fibers in several layers of the main olfactory bulb, namely, glomerular layer, mitral cell layer, internal plexiform layer, and granule cell layer. Soria-Gómez et al. (2014) showed that CB_1_ is abundantly expressed on axon terminals of centrifugal cortical glutamatergic neurons that project to inhibitory granule cells in the granule cell layer. Wang et al. (2019) showed that CB_1_ staining was tightly restricted to neuron-like processes in the glomerular layer (Figure 3A). As a control, this staining was absent in the same regions of the main olfactory bulb taken from CB_1_^−/−^ mice (Figure 3B). The staining outlined glomeruli in the MOB, i.e., it was periglomerular in nature as demonstrated by co-staining with recoverin (Wang et al., 2019). No pronounced staining was observed in the external plexiform or mitral cell layer (Figure 3C), except for rare processes in the external plexiform layer. The staining pattern established the presence of CB_1_ in the glomerular layer. Freundt-Revilla et al. (2017) reported that despite a lack of immunostaining in the mitral cell layer, mitral cell axons were moderately CB_1_ positive, suggesting that targets of main olfactory bulb output neurons could be under CB_1_ regulation.

The identity of the CB_1_-expressing neuronal cell type was identified by testing CB_1_ staining against markers of interneuron populations using tissue from GAD67-GFP reporter mice (e.g., Figure 1A) or an antibody against GAD65. CB_1_ colocalized with a small subset of GAD65-positive interneurons (Figure 3D). The CB_1_ staining was restricted to neuronal processes and did not include neuronal cell bodies. Knockout controls combined with immunohistochemistry staining support the observation of CB_1_ expression in periglomerular cells.

Measurements of eCBs in the main olfactory bulb yielded evidence for the presence of CB_1_ agonist 2-AG (Soria-Gómez et al., 2014; Wang et al., 2019). Likewise, other related lipids were detected in the mouse main olfactory bulb (Wang et al., 2019). In the main olfactory bulb, 2-AG levels were the highest among those tested, consistent with its hypothesized role as a CB_1_ receptor ligand (Wang et al., 2019). AEA levels were lower which was consistent with findings for other regions of the brain (Cravatt et al., 2001). Cannabinoid and related lipid levels were at the low end of the spectrum of values reported for the brain, but this may be consistent with the highly restricted expression of CB_1_. While 2-AG is thought to more relevant for CB_1_ signaling in neurons (Straiker and Mackie, 2005, 2009; Straiker et al., 2009; Tanimura et al., 2010; Jain et al., 2013), recent evidence also points to roles for AEA (e.g., Puente et al., 2011).

Many of the identified components of cannabinoid signaling are present in the main olfactory bulb of the mouse (Wang et al., 2019). mRNA expression of a wide range of cannabinoid-related proteins (CB1, CB2, NAPE-PLD, ABHD4, GDE1, FAAH, NAAA, DGLα, DGLβ, MGL, ABHD6, and ABHD12) was tested in the mouse main olfactory bulb with RT-PCR. High levels of CB1 mRNA were present, while the level of CB2 mRNA was very low. The enzymes involved in AEA and 2-AG biosynthesis (e.g., NAPE-PLD, ABHD4, GDE1 for AEA; DGLα/β for 2-AG) and metabolism (e.g., FAAH and NAAA for AEA; MGL and ABHD6/12 for 2-AG) were almost all present in the mouse main olfactory bulb, although the expression of MGL mRNA was relatively low compared with ABHD6/12. When the gene for MGL is deleted, 2-AG increases strongly in the brain of the mouse (Pan et al., 2011). Other enzymes can metabolize 2-AG, such as ABHD6 and ABHD12 (Blankman et al., 2007), possibly in a complementary manner that depends on the brain region or neural circuit. It is not clear if MGL mRNA expression corresponds to protein levels. Overall, the available immunohistochemistry and biochemical data indicate that the mouse main olfactory bulb is well supplied with known and hypothesized enzymes for the synthesis/metabolism of AEA and 2-AG.

Release of endocannabinoids in the main olfactory bulb is thought to occur from several cell types, including external tufted cells and mitral cells as well as GABAergic cells in the granule cell layer, namely, deep short-axon cells and granule cells (Heinbockel and Wang, 2015; Heinbockel et al., 2016; Freundt-Revilla et al., 2017; Pouille and Schoppa, 2018; Zhou and Puche, 2021). Endocannabinoids regulate neuronal activity and signaling in glomerular cells (Wang et al., 2012, 2019; Heinbockel et al., 2016; Pouille and Schoppa, 2018) and corticofugal input to the main olfactory bulb (Soria-Gómez et al., 2014; Pouille and Schoppa, 2018; Zhou and Puche, 2021).

## CB_1_-Mediated Depolarization-Induced Suppression of Inhibition (DSI)

Our knowledge of eCB signaling in glomerular circuits and the relevance of CB_1_ for output neuron activity in main olfactory bulb glomeruli is limited. Since recent work demonstrated cannabinoid levels and the expression of CB_1_ and other genes associated with cannabinoid signaling in the main olfactory bulb, it was likely that agonists/antagonists of CB_1_ have a functional effect on cellular and network activity of key neuronal cell types, periglomerular cells, tufted cells, and mitral cells, in a slice preparation of the mouse main olfactory bulb (Wang et al., 2012, 2019). DSI had not been demonstrated in the olfactory system until a few years ago. Results obtained in periglomerular cells established that DSI is present in the glomerular layer of the main olfactory bulb. Periglomerular cells form inhibitory GABAergic dendrodendritic synapses with external tufted cells. In turn, external tufted cells form excitatory glutamatergic dendrodendritic synapses with periglomerular cells. In mouse brain slices, cannabinoids display strong, direct inhibitory effects on periglomerular cells and weak effects on external tufted cells (Wang et al., 2012). When external tufted cells are depolarized by injecting single electrical pulses or a train of pulses of depolarizing current, the inhibitory postsynaptic currents (IPSCs) are transiently suppressed which suggests the presence of retrograde endocannabinoid signaling, namely, DSI in external tufted cells. External tufted cells display intrinsic bursting of action potential which is mediated by several of their intrinsic conductances (Liu and Shipley, 2008). Burst firing of external tufted cells is thought to trigger the release of eCBs which in turn directly inhibit periglomerular cells and reduce their GABA release. This is evident as a transient reduction of periglomerular cell inhibitory input (IPSCs) to external tufted cells (Wang et al., 2012). The presence of DSI in external tufted cells depends on voltage step duration and step number. With a step duration of one second, external tufted cells do not show clear DSI. With a step duration of five seconds, transient DSI is evoked. Furthermore, a train of depolarizing voltage steps (>3) strengthens the inhibition of IPSCs. This suggests that excitation of external cells in the form of rhythmic bursting triggers the release of eCBs and, thereby, regulates glomerular activity. Bursting of neurons is present in other brain systems as well, and bursting may modulate eCB release also in those neurons and not only in the main olfactory bulb.

The electrophysiological evidence indicates that the eCB system plays a functional role in regulating neuronal activity and signaling in main olfactory bulb glomeruli through CB_1_-mediated retrograde signaling and control of excitability among glomerular neurons in the form of DSI. External tufted cells receive monosynaptic olfactory sensory nerve input. The inhibitory effect of CB_1_ on periglomerular cells by eCBs reduces inhibitory input to external tufted cells and could enhance external tufted cell sensitivity to weak sensory inputs by depolarizing the membrane potential closer to the spike threshold. Periglomerular cells presynaptically inhibit olfactory afferent input in the glomerular layer of the main olfactory bulb (Shipley and Ennis, 1996; Keller et al., 1998; Hsia et al., 1999; Wachowiak and Cohen, 1999; Aroniadou-Anderjaska et al., 2000; Berkowicz and Trombley, 2000; Ennis et al., 2001, 2007; Palouzier-Paulignan et al., 2002; Murphy et al., 2005; Ennis et al., 2007). CB_1_-mediated inhibition of periglomerular cells could also reduce inhibition of presynaptic olfactory nerve terminals and increase their glutamate release. In this way, activation of CB_1_ on periglomerular cells could increase the overall sensitivity of glomerular neurons to sensory inputs.

## CB_1_ Agonists and Antagonist Modulate Mitral Cell Activity Through Periglomerular Cells

**Figure 4:**
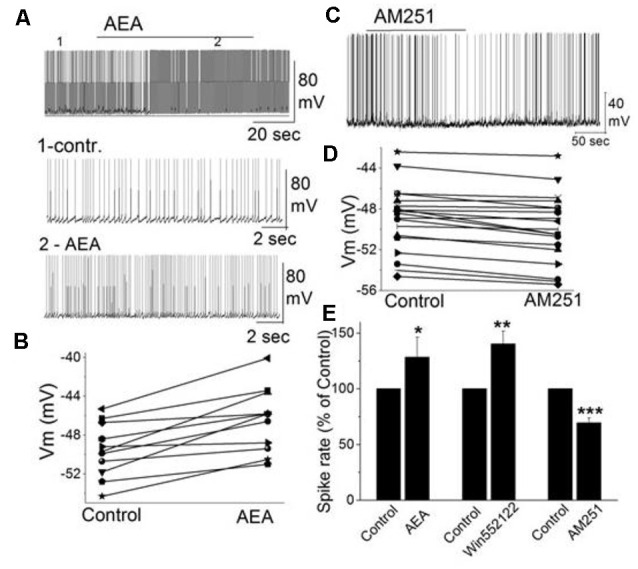
The activity of mitral cells was regulated by cannabinoids. **(A)** Original recording illustrates the increased firing rate of a mitral cell in response to bath application of CB_1_ agonist AEA (10 μM). Time points 1 and 2 in the upper trace are shown at higher time resolution in the second and third trace, resp. **(B)** Representative mitral cell depolarized by AEA (10 μM). **(C)** Original recording from a mitral cell displayed the reduction in firing rate and hyperpolarization following application of CB_1_ antagonist AM251. **(D)** Representative mitral cell with hyperpolarized membrane potential in response to AM251. **(E)** Group data of the effect of CB_1_ agonists and antagonist AM251 on spike rate. Asterisks indicate significance level (*p < 0.05, **p < 0.01, ***p < 0.001). From Wang et al. (2019).

In addition to external tufted cells and periglomerular cells, mitral cells as the key main olfactory bulb output neurons are also regulated through CB_1_, even though, this is through an indirect mechanism. Mitral cells exhibit a background action potential firing rate ranging from 1 to 8 Hz (Heinbockel et al., 2004). The selective CB_1_ agonist anandamide increases mitral cell firing rate and depolarizes the membrane potential (Figures 4A,B; Wang et al., 2019). Similar excitatory effects on mitral cell firing rate are seen to CB_1_ agonists WIN 55,212-2 mesylate (WIN552122, WIN), and CP 55,940. The selective CB_1_ antagonists AM251 hyperpolarizes mitral cells and reduces their firing rate (Figures 4C–E). Pretreating mitral cells in acute brain slices with AM251 prevents WIN from increasing the mitral cell firing rate or changing the membrane potential, indicating that CB_1_ is involved in cannabinoid-mediated modulation of mitral cell activity.

Spontaneous GABAergic inputs from periglomerular cells to mitral cells might be the target of CB_1_-mediated modulation (Wang et al., 2012; Harvey and Heinbockel, 2018). The electrophysiological and anatomical data described above are consistent with CB_1_-mediated modulation of periglomerular GABAergic interneurons. Inhibitory synaptic transmission originating from GABAergic interneurons such as periglomerular cells could modulate mitral cell activity. In mitral cells, bath application of AM251 increased the frequency of spontaneous IPSCs (sIPSCs) and evoked outward currents which is consistent with the inhibitory effect of AM251 on mitral cells (Figures 4C,D; Wang et al., 2019). In contrast, CB_1_ agonist WIN reduced the sIPSC frequency in mitral cells and evoked inward currents when AMPA and NMDA receptors are blocked (CNQX, AP5). The results can be interpreted such that cannabinoids synaptically regulate mitral cell activity by regulating GABA release from interneurons.

Potentially, one of several types of GABAergic interneurons in the main olfactory bulb can be the target of direct regulation by CB_1_. Periglomerular cells are likely candidates for direct effects of cannabinoids since CB_1_ is robustly expressed in these cells (Wang et al., 2019). As described above, a CB1 agonist inhibits periglomerular cells, whereas a CB_1_ antagonist activates them (Wang et al., 2012). This is the inverse response pattern to CB_1_ activation compared with mitral cells (Figure 4). These findings suggested that CB_1_ indirectly regulated mitral cell activity by modulating inhibitory inputs to mitral cells. Granule cells form another population of GABAergic interneurons in the main olfactory bulb and are known to regulate mitral cell activity through dendrodendritic synapses (Shepherd et al., 2004). In a subglomerular slice preparation, the olfactory nerve layer and glomerular layer are removed (Dong et al., 2007). After removal of the glomerular layer, it is possible to determine if granule cells or periglomerular cells play a role in CB_1_ mediated mitral cell regulation. Mitral cell properties in subglomerular slices (*V*m, input resistance, spike rates) are not different from mitral cells in intact main olfactory bulb slices. However, in subglomerular slices, a CB_1_ agonist fails to depolarize mitral cells or change the frequency of spiking or the membrane potential. Similarly, a CB_1_ antagonist fails to decrease the frequency of mitral cell spiking or change the membrane potential in subglomerular slices. The results indicate the glomerular layer and, specifically, periglomerular cells, as being involved in CB_1_-mediated mitral cell modulation and rule out granule cells as modulators of mitral cell activity through CB_1_ activation (Wang et al., 2019).

**Figure 5:**
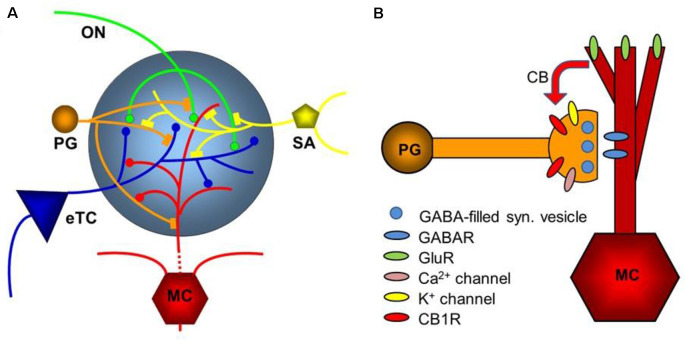
Diagram of the glomerular network. **(A)** Olfactory nerve (ON) afferents enter the main olfactory bulb through the olfactory nerve laver to synapse with periglomerular cells (PG), mitral cells (MC), and tufted cells (of which only external ones, eTCs, are shown) within the glomerular layer. Periglomerular cells inhibit olfactory nerve terminals, external tufted cells, and mitral cells. The processes of Short Axon (SA) cells, which are GABAergic and dopaminergic, receive excitatory synaptic input and form extensive interconnections between glomeruli. Mitral cell apical dendrites convey sensory information to deeper layers of the main olfactory bulb. Mitral cells and tufted cells form dendrodenritic synapses with glomerular neuronal processes. (**B)** Dendrodendritic interactions of mitral cells and periglomerular cells. Cannabinoids are released non-synaptically by mitral and potentially other cells and act on cannabinoid receptors in periglomerular cells to modulate their synaptic release of GABA. Only the apical dendrite of the mitral cell is shown. GABAR—GABA receptors, GluR—ionotropic and metabotropic glutamate receptors. Panel **(A)** is modified from Harvey and Heinbockel (2018) with the permission of the publisher MDPI. Panel **(B)** is from Wang et al. (2019).

The key finding described above is that activity of mitral cells and external tufted cells, output neurons of the main olfactory bulb, is regulated in a CB_1_-dependent manner by a periglomerular interneuron network, likely based in a small subset of GAD65-positive GABAergic interneurons (Figure 5; Wang et al., 2019). This offers additional evidence that olfactory sensory inputs to the brain are modulated by the cannabinoid signaling system. Experiments with subglomerular slices indicate that: (a) CB_1_-mediated effects are limited to a glomerular circuit that does not involve granule cells; and (b) eCB-mediated regulation involves apical dendrites of mitral cells (Figure 5). The components of this cannabinoid signaling circuit regulate the activity of the main output neurons. Activation of CB_1_ in this circuit relieves the interneuron-mediated inhibition and may render mitral cells and other output neurons more responsive to odor stimulation and synaptic input.

## Regulation of Olfactory Neurons in a CB_1_-Dependent Manner

Different signaling pathways may contribute to CB_1_ modulation. One pathway extends from periglomerular cells to mitral cells and external tufted cells. In another pathway, CB_1_ may indirectly regulate glutamate release from olfactory nerve terminals by reducing presynaptic inhibition of glutamate release. This hypothesis suggests that CB_1_ regulates mitral cell activity, namely from GABAergic glomerular cells to olfactory nerve terminals to mitral cells. This hypothesis received support by the observation that the CB_1_ antagonist AM251 increased the frequency of sIPSCs in mitral cells (Wang et al., 2019). Endocannabinoids that are released in the glomerular layer inhibit periglomerular cells to reduce their GABA release. This relieves presynaptic inhibition of olfactory nerve afferents. Consequently, mitral and external tufted cells show stronger glutamate-mediated excitation.

Other studies have established additional functional consequences of CB_1_-mediated regulation in the main olfactory bulb. Cannabinoid signaling in the main olfactory bulb can regulate appetite and adjust the olfactory threshold through centrifugal fiber input to inhibitory granule cells as a means of cortical feedback to the main olfactory bulb (Soria-Gómez et al., 2014; Pouille and Schoppa, 2018; Terral et al., 2020). In a key article, showed that feeding behavior can be regulated by eCBs in the olfactory system such that CB_1_ increases feeding behavior in fasted mice (hyperphagia) through enhanced detection of food, mediated by olfactory mechanisms (Soria-Gómez et al., 2014). CB_1_ is expressed in the terminals of corticofugal glutamatergic projections to the granule cell layer in the main olfactory bulb. Endocannabinoids and exogenous cannabinoids activate CB_1_ and subsequently, promote increased odor detection and feeding after fasting. This CB_1_-mediated regulation of feeding behavior is accomplished through olfactory corticofugal circuits such that excitatory drive from olfactory cortical areas to the main olfactory bulb is reduced. The authors have demonstrated these functional consequences of CB_1_-mediated regulation in the main olfactory bulb and link hunger, olfaction, and feeding behavior to an eCB-mediated neuromodulation mechanism of synaptic transmission in the main olfactory bulb that relies on CB_1_-dependent control of cortical feedback to olfactory circuits (Soria-Gómez et al., 2014).

The relevance of centrifugal or feedback projection from higher-order olfactory areas to the main olfactory bulb for circuit dynamics and sensory processing has been demonstrated in other studies as well (Boyd et al., 2012; Rothermel and Wachowiak, 2014; Mazo et al., 2016; In ’t Zandt et al., 2019; Zhou and Puche, 2021). Centrifugal fibers add another level of regulatory control through CB_1_. Pouille and Schoppa (2018) examined the role of the eCB system in regulating centrifugal input to the main olfactory bulb. They observed that CB_1_ mediates widespread suppressive effects on synaptic transmission at centrifugal fiber synapses onto interneurons in the main olfactory bulb and can bidirectionally change the ratio of inhibition and disinhibition of mitral cells depending on circuit activation through its effects on centrifugal fibers. Their results demonstrate that eCBs regulate excitatory corticofugal input to deep short axon cells and granule cells in the main olfactory bulb.

In addition to the robust eCB modulation of excitatory inputs to granule cells, there is also eCB modulation of the inhibitory cortical inputs to granule cells (Zhou and Puche, 2021). CB_1_ is expressed in the granule cell layer and eCBs are released in this layer. GABAergic neurons in the horizontal limb of the diagonal band of Broca (HDB) project to granule cells in the main olfactory bulb. The authors demonstrate that GABAergic projections of the HDB are tonically activated by eCBs and inhibit granule cells, similar to eCB-mediated modulation of glutamatergic projections to granule cells (Pouille and Schoppa, 2018). This modulation of inhibitory synaptic input has the potential to regulate the balance of cortical feedback excitation and inhibition of granule cells. In turn, the GABAergic output of granule cells can change and affect the main olfactory bulb output to higher-order olfactory processing areas. Since mitral cells project to higher olfactory processing areas in the cortex, activation of inhibitory GABAergic projections to granule cells could result in increased excitability of mitral cells through the disinhibition of granule cell inhibitory action on mitral cells (Zhou and Puche, 2021).

**Outlook**: During the past two decades, the endocannabinoid system has emerged as an important neuromodulatory system. In the main olfactory bulb, neurons express CB_1_, but our understanding of its cellular, network, and behavioral function remains in its infancy. Behavioral correlations are difficult to establish but are needed to achieve an integrated understanding of the role of the endocannabinoid system for olfactory processing. The work by Soria-Gómez et al. (2014) remains the only study of the olfactory system that has placed the endocannabinoid system in a behavioral context by linking an internal metabolic state (hunger) to sensory perception and subsequent behavior, namely food intake. As discussed above, the authors reported that CB_1_ receptor-dependent control of excitatory drive from centrifugal feedback projections to the olfactory bulb determines the efficiency of olfactory processes and food intake in fasted mice. In contrast to the work on cannabinoid signaling in the glomerular input region (Wang et al., 2012, 2019), the study by Soria-Gómez et al. (2014) focused on neural processes deeper in the olfactory bulb, primarily involving those olfactory bulb neurons (granule cells) that receive heavy CNS feedback rather than direct sensory input from the nasal olfactory epithelium. The authors took advantage of the structural organization of the main olfactory bulb by integrating three separate neural components in their experiments: sensory (olfactory) input, central processing in the main olfactory bulb, and behavioral output in terms of feeding in the overall framework of the internal state of the animal (hunger). The authors emphasized the relevance of cortical feedback to the olfactory bulb as a means to control odor detection. The study clearly established the relationship between food intake and olfactory processing and implicates the endocannabinoid system as a key player in this signaling pathway. As such, their study opened the door for future studies and follow-up questions to reveal the mechanisms of endocannabinoid signaling in the olfactory system. The authors found a THC effect on both olfactory detection thresholds and habituation, while the latter effect had no correlation to food intake. While the authors suggested that the “enhancement of olfactory detection is likely the main mechanism linking (endo)cannabinoid signaling in the olfactory bulb to increased food intake,” it is not clear if that is the case or if there are other underlying mechanisms. Future studies that change the odor concentration in the environment might show if feeding behavior is indeed affected by odor intensity. This issue might be confounded by the fact that high levels of odor input might have an aversive effect on eating. In this context, future studies might ask about the role of the endocannabinoid system in non-fasting animals. Is it only during the sensation of hunger that endocannabinoids play a role in this circuit or do cannabinoids have other, possibly homeostatic functions.

Soria-Gómez et al. (2014) postulated that by reducing overall granule cell-mediated inhibition of mitral cells, mitral cells become more sensitive, and that would lower the odor detection threshold. While this is plausible, the actual mechanism for lowering the odor detection threshold remains to be determined. Other cellular mechanisms could come into play. These mechanisms could work in the peripheral input region of the main olfactory bulb rather than in the deeper granule cell layer.

Recent work focused on centrifugal glutamatergic and GABAergic input to the main olfactory bulb and its cannabinoid modulation (Pouille and Schoppa, 2018; Zhou and Puche, 2021). This is not the only centrifugal input that reaches the main olfactory bulb. Rather, other areas of the brain also provide feedback cortical projections with cholinergic, dopaminergic, serotonergic, or noradrenergic input. It remains to be determined if this input is also subject to regulation by cannabinoids.

CB1 knockout mice have been valuable tools in delineating the role of cannabinoid signaling in the nervous system. Likewise, experiments that selectively activate or eliminate olfactory processing channels coming either from the periphery or centrifugal fibers could be critical in understanding the role of this signaling system in a behavioral context.

## Neural Circuitry in The Retina

The light must pass through several anterior eye structures before it reaches the light-sensing retina at the back of the eye. The retina, with similar embryonic origins as the brain, is made up of three neuronal layers (Ramon y Cajal, 1891) depicted schematically in Figure 1B. Because the retina receives little in the way of centrifugal neuronal inputs from the CNS, much of the pre-processing of the complex visual stimulus must be done within the retina. This requires contributions from 50 or more distinct neuronal types (Röhrenbeck et al., 1987; Ghosh et al., 2004). The most numerous of these are the photoreceptors—the low-light sensing rods and the bright-light color-sensing cones—that are located at the outermost layer of the retina with their light-sensing apparatus facing away from the light (Rodieck, 1998). The light, therefore, passes through the transparent retina before commencing a journey forward through two neuronal layers and then back again to the brain (Ramon y Cajal, 1891). The photoreceptors convert their light response to a chemical signal, a change in the release of glutamate that is released into the outer synaptic or plexiform layer (OPL). The second-order neurons of the outer nuclear layer include bipolar cells, horizontal cells, and amacrine cells (Ramon y Cajal, 1891). Bipolar cells come in two classes, the rod bipolar cells that are stimulated by the rod photoreceptors in dim light and the cone bipolar cells that are activated under bright-light conditions. Seen most simply, bipolar cells receive inputs from photoreceptors and deliver outputs to ganglion cells in the next synaptic layer, the inner plexiform layer (IPL; Rodieck, 1998). The IPL is highly layered and many of the neuronal projections into this region are restricted to defined layers (Ramon y Cajal, 1891). Rod bipolar cells are represented by a single type of neuron while cone bipolar cells come in multiple forms. The mouse retina, for instance, has at least nine different types of cone bipolar cells that differ in their lamination, morphology, and likely their function (Ghosh et al., 2004). Horizontal cells adjoin the OPL, where they help to integrate and regulate the photoreceptor outputs. Amacrine cells come in numerous forms and are distinguished by the lack of an axon (Ramon y Cajal, 1891). Amacrine cells play varied roles, and some form part of the rod signaling pathway, but in general, their job is to modulate the output of bipolar cells and the inputs of the final element of the retinal signaling pathway: ganglion cells (Masland, 2012). Ganglion cells line the innermost layer of the retina. Their dendrites extend into the IPL, generally with distinct lamination, while their axons project into the brain *via* the optic nerve. Estimates of the number of different kinds of ganglion cells vary but may exceed 30 (Sanes and Masland, 2015) and often exhibit distinct firing properties in response to specific visual stimuli (Grünert and Martin, 2020). Broadly speaking then, the signaling pathway within the retina begins with photoreceptors, then passes through bipolar cells and then ganglion cells, with the signal modified by complex circuit contributions from horizontal and amacrine cells. The ganglion cells are the output cells of the retina and send their axons out of the eye through the optic nerve to the lateral geniculate nucleus of the thalamus. There ganglion cell axons synapse onto neurons that project to the visual cortex.

## Expression of CB_1_ and Cannabinoid Enzymatic Machinery in Retinal Circuits

Whatever the true origins of cannabis, it is intriguing that Shen Nung referred to ostensible visual effects of the plant, indicating that the plant could make one “see devils”. Hallucinations are not among the classical outcomes of cannabis consumption but have been reported at higher concentrations (Perez-Reyes et al., 1972). The concentrations required to achieve the desired effects of cannabis (chiefly euphoria) are lower than those for explicitly hallucinatory effects (Perez-Reyes et al., 1972; Hollister and Gillespie, 1973). It may be for this reason that the plant has not been associated with visual effects in modern times. And the visual effects that have been reported were typically ascribed to cortical and hypothalamic actions akin to those of the serotonin-receptor activating psychedelics LSD and psilocybin, rather than being retinal in nature.

There was therefore little reason to think that cannabinoids played much of a role in the eye until Hepler’s groundbreaking work in the 1970s (Hepler and Frank, 1971), linking cannabinoid use to a lowering of intraocular pressure. Though the consequence of glaucoma is ultimately on retinal function, the reasonable presumption was that the site of action was in the anterior eye. Though dedicated endocannabinoid receptors were identified in the early 1990s followed by the components of a general cannabinoid signaling system, there remained very little reason to expect that such a system was functional in the retina. Some anecdotal reports of visual effects appeared in the scientific literature (Russo et al., 2004), most curiously two reporting the claims of Jamaican fishermen that smoking cannabis enhanced their night-vision (Reese, 1991; West, 1991), however, these anecdotes are difficult to interpret, especially given that another largely anecdotal report describes a dimming of vision (Consroe et al., 1997). In the 1990s, the field began to see experimental investigations as well as descriptive reports of receptor expression. The first study that examined retinal CB_1_ expression concluded that CB_1_ was not present (Galiègue et al., 1995) but several subsequent studies appeared to counter this (Buckley et al., 1998; Porcella et al., 1998, 2000; Straiker A. J. et al., 1999; Straiker A. et al., 1999).

**Figure 6:**
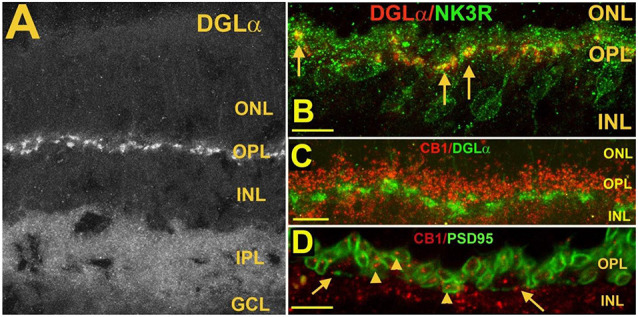
Postsynaptic diacylglycerol lipase α (DGLα) in Type 1 OFF cone bipolar cells, is juxtaposed to presynaptic CB1 in photoreceptor terminals.** (A)** DGLα staining in the mouse retina. **(B)** DGLα (red) colocalizes with post-synaptic terminals of Type 1 OFF bipolar cells, as marked by NK3R (green, arrows). **(C)** Flattened Z-series indicates CB_1_ (red) closely juxtaposes distal to DGLα (green), consistent with rod spherule and cone pedicle localization. **(D)** CB_1_ (red) costaining with PSD95, which outlines rod spherules (arrows) and cone pedicles (angled arrows) shows CB_1_ present within terminals of both rod and cone photoreceptor terminals. Scale bars: **(A)**: 50 μm; **(B–D)**: 25 μm. Adapted from Hu et al. (2010).

CB_1_ receptors are located chiefly in the two plexiform layers (Hu et al., 2010). In the outer plexiform layer CB_1_ staining is seen in both rod and cone terminals (Figures 6C,D; Straiker A. et al., 1999; Hu et al., 2010). In the IPL, CB_1_ staining is diffuse and present in both ON and OFF zones, with little sign of lamination. Yazulla et al. (2000) reported CB_1_ staining in rat rod bipolar cells stained with protein kinase C, a kinase that labels rod bipolar cells (Negishi et al., 1988). Wang et al. (2016) reported INL CB_1_ expression in rod bipolar cells, several populations of cone bipolar cells as well as AII amacrine and GABAergic amacrine cells. Neither study made use of CB_1_ knockout controls [mouse and rat CB1 receptors differ by only one residue (Matsuda et al., 1990)]. In contrast, Zabouri et al. (2011) reported CB1 expression in the rat in nearly all amacrine cells, as well as horizontal cells and most ganglion cells but not in rod bipolar cells. Other studies have examined species such as primate (e.g., Bouskila et al., 2012). The conflicting results from studies in the rat are difficult to parse. An additional follow-up study of CB1 expression in the IPL of the mouse with knockout controls would therefore be welcome.

Based on the expression pattern of CB1 and what is known of CB1 function in neurons, forms of cannabinoid-mediated plasticity that involve only neurons are likely to consist of two or three forms of retrograde signaling: (1) a retrograde signal between cells in the internal nuclear layer (INL) and the photoreceptors (see schematic Figure 9); (2) an INL circuit between postsynaptic ganglion cells and bipolar and/or amacrine inputs, and possibly; and (3) a circuit between neurons in the INL. More specifically, in the OPL, where the CB_1_ staining is clearly seen in photoreceptor terminals (Straiker A. et al., 1999; Straiker and Sullivan, 2003; Hu et al., 2010), cannabinoid signaling is likely to be retrograde at these terminals, i.e., either bipolar or horizontal cells in the INL release endocannabinoids onto presynaptic CB_1_ receptors. Ordinarily, this would be expected to inhibit neurotransmitter release, though the inverse relationship between calcium and glutamate release in photoreceptors complicates this simple view.

**Figure 7:**
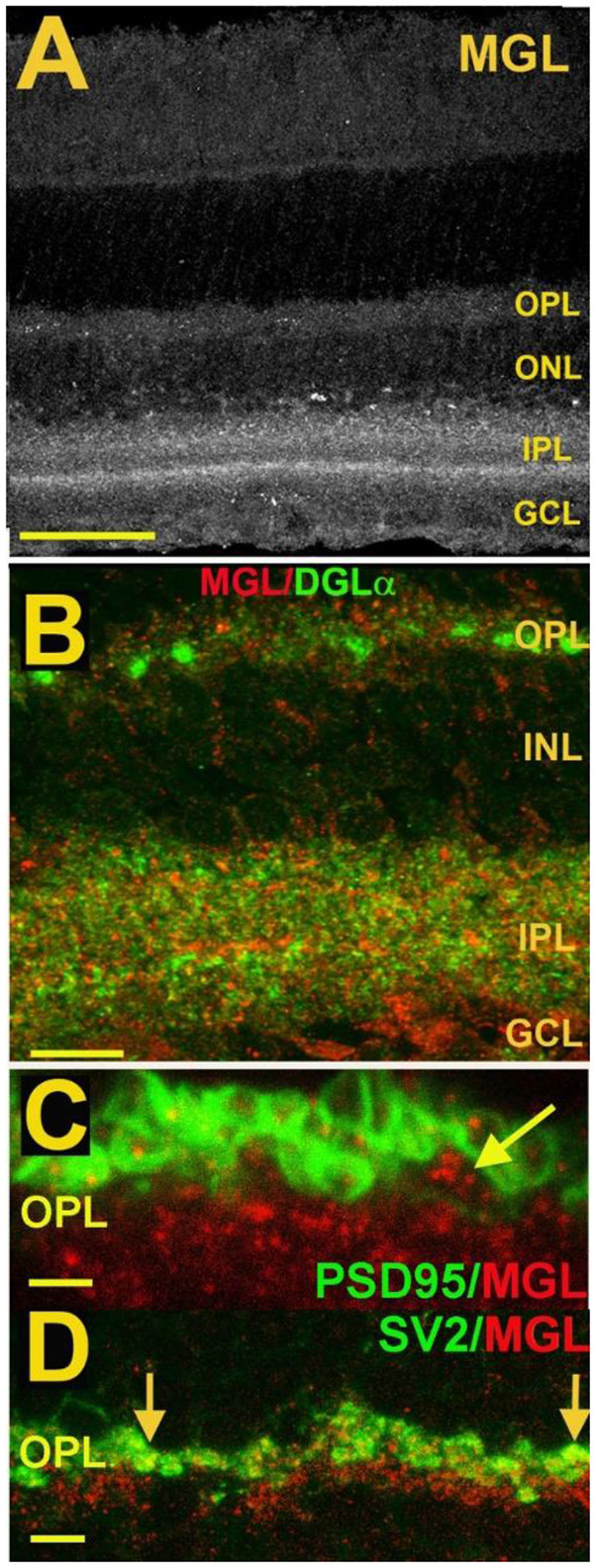
MGL is present in rod spherules and cone pedicles in the OPL and is expressed prominently in two laminae of the IPL.** (A)** MGL staining in the mouse retina.** (B)** MGL (red) staining with DGLα (green) shows their apparent non-overlap and relative localization. **(C)** MGL (red) costaining with PSD95 (green), a marker that outlines rod and cone terminals shows MGL within a cone terminal (arrow) as well as punctate staining within multiple rod spherules. **(D)** MGL (red) costaining with SV2 (green), a marker for rod spherules, shows substantial overlap in the OPL (arrows). Scale bars: **(A)**: 60 μm; **(B)**: 25 μm; **(C)**: 15 μm; **(D)**: 25 μm. Adapted from Hu et al. (2010).

**Figure 8:**
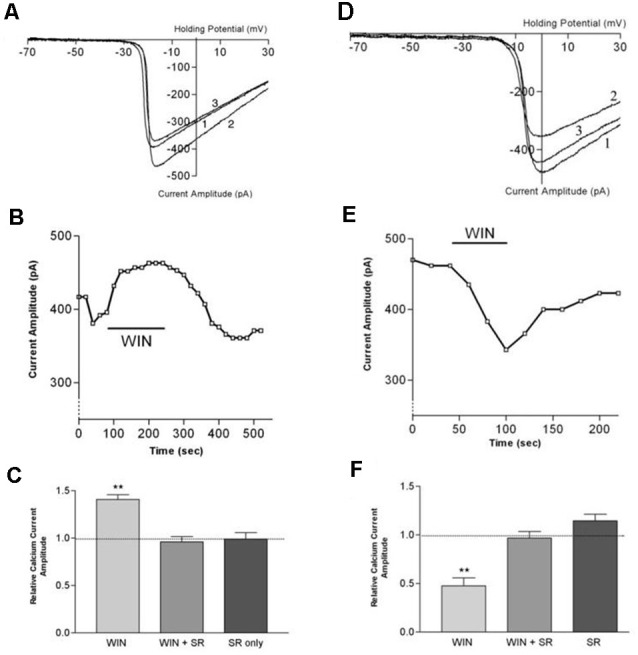
CB1 receptor activation differentially affects ICa in rod vs. cone photoreceptors. **(A)** I_Ca_–V curves before application of WIN 55,212-2 (WIN, 1), after application (2), and after wash (3). **(B)** Peak amplitude of the calcium currents from the same cell over time, indicating a robust enhancement followed by gradual recovery. **(C)** Summary of results with WIN alone, WIN combined with the selective CB1 receptor antagonist SR141716A and SR 141716A alone. **(D)** I_Ca_–V curves before application of WIN 55,212-2 (1), after application (2), and after wash (3). **(E)** Peak amplitude of the calcium currents from the same cell over time indicating a robust inhibition followed by gradual recovery. **(F)** Summary of results with WIN alone, WIN plus the selective CB1 receptor antagonist SR141716A, and SR 141716A alone. **P < 0.01, Student’s *t*-test. Adapted from Straiker and Sullivan (2003).

**Figure 9:**
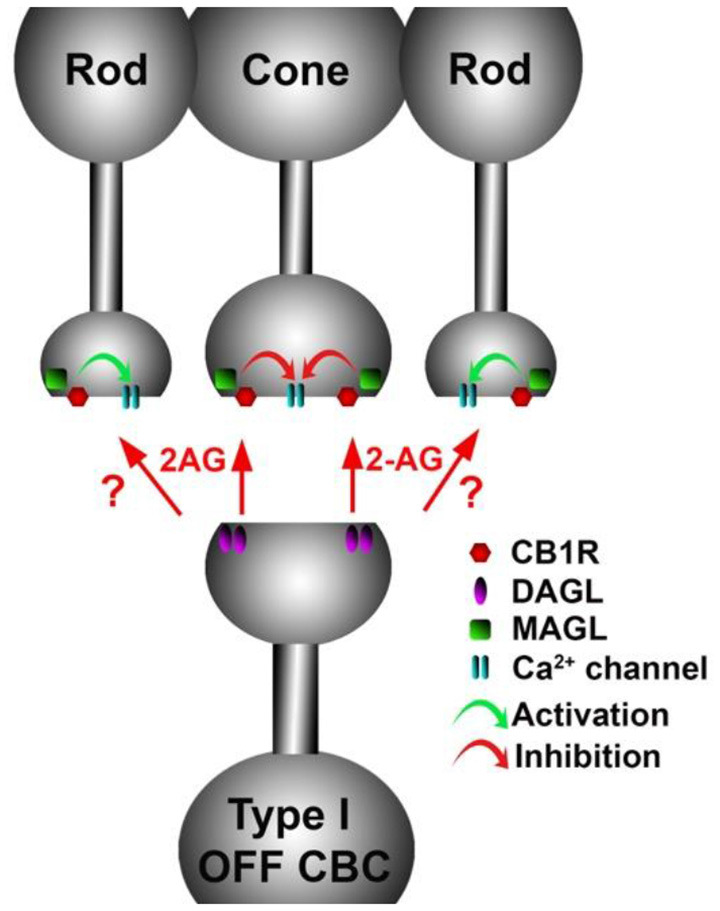
A retrograde cannabinoid CB1 circuit in the OPL. Rod and cone photoreceptors signal to neurons in the inner nuclear layer (INL). Both rods and cones express CB_1_ receptors. The 2-AG synthesizing enzyme diacylglycerol lipase (DAGL) is present in the post-synaptic terminal of Type I OFF cone bipolar cells (OFF CBC). 2-AG released by DAGL travels retrogradely, presumably to CB1 receptors on cones, and perhaps also rods. CB_1_ receptors, when activated, differentially act on presynaptic calcium channels, activating them in cones and inhibiting them in rods.

The expression of endocannabinoid synthesizing enzymes may offer some insight into the circuitry since, as noted previously, the 2-AG synthesizing enzyme DAGLα is typically postsynaptic. The relative absence of DAGLβ [excepting blood vessel-associated expression (Hu et al., 2010)] probably leaves the field to DAGLα. In the outer plexiform layer (OPL), DAGLα expression is highly specific to a type of cone bipolar cell (Hu et al., 2010; Figure 6). This strongly suggests that in the mouse a retrograde circuit exists between cone bipolar cells and cone photoreceptors. Consistent with this, the 2-AG metabolizing enzyme MGL is seen in both rod and cone terminals (Figure 7; Hu et al., 2010). The prominent expression of DAGLα in cone bipolar cells leaves CB1 in rod terminals without a “dance partner” though the presence of MGL in rod terminals underscores a likely 2-AG role. It is possible, given the known ability of cannabinoids to spread (Wilson and Nicoll, 2001), that 2-AG released by cone bipolar cells acts on both rods and cones. Alternatively, CB_1_ on rod bipolar cells may be targeted by anandamide from an unknown source. A final possibility is that expression patterns for both receptors and relevant enzymes may vary diurnally. FAAH levels cycle diurnally in the eye, regulating the cycling of ocular pressure in the anterior eye (Miller et al., 2016).

To further complicate the picture, metabotropic suppression of excitation/inhibition (MSE or MSI) can involve any number of G_q_-coupled GPCRs that, when activated, cause the postsynaptic release of 2-AG. “Classical” MSE involved Group I mGluR and M1/M3 muscarinic receptors (Maejima et al., 2001; Kim et al., 2002). However, since MSE can in principle occur *via* activation of any postsynaptic G_q_-coupled metabotropic receptor, it is possible that the actual circuit of retrograde inhibition is due to transmitter release from a separate upstream neuron. That neurotransmitter could at a minimum be acetylcholine, serotonin, substance P, or orexin (e.g., Haj-Dahmane and Shen, 2005; Drew et al., 2009).

There are conflicting reports regarding a potential role for CB2 in retinal function (e.g., Borowska-Fielding et al., 2018; Cecyre et al., 2020) but since there is little evidence for a CB2 role in the olfactory system, we will focus on CB1 receptor anatomy and function.

## Cannabinoid Function in The Retina—Early Studies

The first evidence of a potential cannabinoid-receptor-based functional effect in the retina appeared in 1996 with Schlicker et al. reporting that cannabinoids lower the production of dopamine in a porcine retinal preparation (Schlicker et al., 1996). The work by Schlicker et al. indicated that cannabinoids inhibit dopamine release in guinea pig retinal discs. Two CB_1_ agonists, WIN55212 and CP55940, gave similar results while the inactive enantiomer of WIN55212 did not. The CB_1_ antagonist SR141716 (rimonabant) blocked this effect and was shown to produce an opposing effect, suggesting the possibility of an endogenous cannabinoid tone regulating dopamine production in the guinea pig retina. Dopamine is released from a restricted subpopulation of tyrosine hydroxylase (TH)-positive amacrine cells that mostly laminate in the distal IPL (Nguyen-Legros et al., 1981). One possibility is that CB_1_ receptors are expressed on these neurons and reduce neurotransmitter release. Alternatively, cannabinoids may inhibit excitatory inputs onto dopaminergic neurons.

## Cannabinoid Function in The Retina—Photoreceptors

Functional studies have described G protein-coupled receptor-dependent modulation of ion channel function in photoreceptors of the tiger salamander by dopamine, adenosine, and somatostatin (Akopian et al., 2000; Stella and Thoreson, 2000; Stella et al., 2002). The calcium currents (I_Ca_) of rods and large single cones respond differentially to activation of the same receptor type. The question arose whether a similar situation might hold for cannabinoid receptors that had been detected at tiger salamander photoreceptor terminals *via* immunohistochemistry (Straiker A. et al., 1999; Straiker and Sullivan, 2003). Straiker and Sullivan (2003) examined the effect of cannabinoid receptor activation on voltage-dependent ion currents in rod and cone photoreceptors of the tiger salamander retinal slice (Straiker and Sullivan, 2003; Figure 8). Studying both I_Ca_ and potassium currents (I_K_) in rods and large single cones, WIN 55212-2 was found to differentially modulates I_Ca_ in rods and cones, enhancing the former and suppressing the latter. In addition, WIN55212-2 inhibited the I_K_ of both rod and large single cone photoreceptors. These actions were blocked by SR141716, indicating that they occurred *via* CB_1_. Calcium modulation is important because calcium is coupled to neurotransmitter release. The modulation occurred *via* protein kinase A (PKA) and L-type calcium channels. These experimental results offered several insights beyond showing cannabinoid responses at the first synapse of the visual system.

The inhibition represented conservation of function—retrograde inhibition of neurotransmitter release by cannabinoids—through an unusual mechanism. Cannabinoid receptors typically inhibit neurotransmitter release *via* βγ inhibition of N- and P/Q-type calcium channels (Mackie et al., 1995). The cannabinoid modulation of L-type calcium channels *via* PKA had been seen before (e.g., Gebremedhin et al., 1999) but not (at the time) to modulate neurotransmitter release.

The cannabinoid modulation of signaling also involved an inverted sign since cannabinoids usually interfere with neurotransmitter release by inhibiting rather than enhancing calcium channel function. A plausible interpretation is a conservation of sign, since glutamate is released constitutively, and the light signal is represented by a decrease in glutamate release. This means that to serve as a retrograde inhibitor, cannabinoids would need to *enhance* glutamate release, which is the case. Lastly, the differential modulation of calcium responses in rods vs. cones suggests that cannabinoids may play opposing roles in terms of regulation of neurotransmission (i.e., suppressing rods while exciting cones or v.v.).

Fan and Yazulla (2005) found that 2 μM WIN55212 inhibited I_K_, I_Cl_, and I_Ca_ in a PKA-dependent and pertussis toxin-sensitive manner. This implied that Gα subunits were inhibiting currents *via* adenylyl cyclase which is supported by others (Straiker et al., 2002). Interestingly at 300 nM and 700 nM the WIN55212 effects were reversed, also in a PKA-dependent manner and also blocked by SR141716 but *not* by pertussis toxin. Pertussis toxin is a reliable blocker of G_i/o_-signaling, suggesting that another pathway might be involved. The inhibition of these currents was occluded by pre-treatment with cholera toxin, which stimulates G_s_ G proteins, thereby raising the possibility that the effect occurred either *via* CB_1_ activation of G_s_ G proteins or perhaps a non- CB_1_ receptor. Glass and Felder (1997) showed that under certain circumstances (i.e., concurrent activation of dopamine D2 and CB_1_ receptors) CB_1_ signals are seen *via* G_s_. The authors offered the possibility that a separate non-CB_1_ receptor might mediate the second G_s_-dependent effect, and this is consistent with the idea of a receptor with shared pharmacology activated by WIN55212. In this study, a ~3-fold concentration difference (700 nM → 2 μM) yielded dramatically different effects *via* the same receptor which merits further study, perhaps with an agonist other than WIN55212.

In a follow-up article, Fan and Yazulla (2004) tested for interactions between CB_1_ and D2 dopamine receptors. D2 activation blocked (or perhaps occluded, since D2 is G_i/o_ coupled) the presumed G_s_-mediated CB_1_ effects on outward currents previously seen with 700 nM WIN55212. This was inconsistent with the findings of Glass and Felder (1997) insofar as one would have expected an even greater G_s_ signaling. In a second finding, the D2 agonist quinpirole, which did modestly inhibit currents at 50 μM, did not do so in an additive manner with WIN55212, suggesting that the inhibitory actions occurred *via* a shared pathway.

In 2006, Struik et al. (2006) reported that WIN55212 altered the cone response to light offset in goldfish retina but since this was not blocked by the CB_1_ antagonist SR141716, this may occur *via* some other target.

Fan and Yazulla (2007) examined retrograde inhibition at the cone-cone bipolar cell interface. Using puffed KCl to depolarize bipolar cells and induce DSE, or a Group I mGluR agonist to induce MSE, the authors provided evidence for retrograde cannabinoid signaling at the first synapse of the visual system. By recording from cone photoreceptors and briefly puffing high-potassium saline onto putative mixed rod/cone bipolar cells, they observed a voltage-dependent potassium current in cones. These responses, consisting of a drop in potassium currents, were altered by several pharmacological interventions. They were blocked by SR141716, suggesting CB1-dependence, but were unaffected by the FAAH inhibitor URB597, arguing against a role for anandamide. A blocker of 2-AG synthesis (tetrahydrolipstatin, THL) did however prevent the response. This argues for a 2-AG rather than an anandamide role in mediating this retrograde signaling, a result that is consistent with general findings for DSE/DSI elsewhere in the CNS. Interestingly, the authors found an effect for COX2 inhibitor nimesulide, consistent with a potential COX2 metabolism of 2-AG that has been reported previously (Kim and Alger, 2004; Straiker and Mackie, 2009; Straiker et al., 2011). Immunohistochemical data from mice (with knockout controls) indicate that MAGL is present in photoreceptor terminals (Figure 7; Hu et al., 2010) and would, therefore, be able to play a role in the breakdown of 2-AG. However, a role for MAGL in OPL cannabinoid signaling remains to be demonstrated. If so, it is possible that MAGL and COX2 act cooperatively as they do in some interneurons. Incidentally, the cooperative activity of MAGL and COX2 account for the faster timecourse of DSI relative to DSE (Straiker and Mackie, 2009; Straiker et al., 2011) and so would be expected to contribute to more rapid 2-AG clearance in photoreceptors.

Fan and Yazulla (2007) also investigated MSE at the same synapse, the cone-bipolar synapse. As noted earlier, this form of retrograde inhibition can be elicited by activation of a post-synaptic G_q_-coupled metabotropic receptor. As mentioned above, early studies were restricted to metabotropic glutamate and muscarinic receptors, and the authors chose mGluR Group I, narrowing the target to mGluR1 with the use of a mGluR5 antagonist (MPEP). In addition, the authors examined the calcium-sensitivity of DSE and MSE. Their findings were that DSE depended on the influx of calcium from external sources through ion channels, whereas MSE depended on intracellular sources of calcium, a result that is broadly consistent with what has been seen for these forms of retrograde inhibition (Kano et al., 2009).

## Cannabinoid Signaling in The Inner Nuclear Layer

The first electrophysiological evidence for a cannabinoid role in retinal signaling derived not from photoreceptors but from bipolar cells, first in L-type calcium currents in bipolar cells of the tiger salamander (Straiker A. et al., 1999) and, subsequently, in potassium currents of the goldfish (Yazulla et al., 2000). The calcium currents likely arise in the axon terminals of bipolar cells and, therefore, represent the action of these receptors in the inner plexiform layer between the inner nuclear layer and ganglion cells.

L-type calcium currents in identified bipolar cells were substantially inhibited by 600 nM WIN55212 and reversed by the antagonist SR141716 (Straiker A. et al., 1999). That study did not examine I_K_ or other currents. Complementary findings were reported for goldfish bipolar cells (Yazulla et al., 2000). Using the other canonical CB_1_ agonist CP55940, the authors found that 1 μM CP55940 inhibited a delayed rectifier potassium current in a population of bipolar cells (“ON Mb”) of the goldfish but not in the presence of SR141716. The authors did not test modulation of I_Ca_ currents in these cells or test I_K_ currents in OFF bipolar cells.

CB_1_/dopamine D1 interactions were studied in ON bipolar cells of the goldfish (Fan and Yazulla, 2005). These cells appear to express both CB_1_ and D1 but not D2 receptors. D1 activation enhanced delayed rectifier potassium currents *via* G_s_ G proteins. Interestingly, the authors found that subthreshold concentrations of WIN55212 did not directly modulate I_K_ currents but nonetheless blocked the D1 potentiation. The effect was blocked by SR141716 and pertussis toxin indicating both CB_1_- and G_i/o_-dependence. The authors proposed that dopamine represents a “light” signal that is opposed by cannabinoids, thereby making cannabinoids a sort of “dark” signal.

More recently Vielma et al. (2020) examined the inhibitory inputs into defined populations of OFF cone bipolar cells in rat retinal slices. They reported that several of these (Types 2, 3a, 3b but not 4) experienced an increase in the frequency of GABAergic but not glycinergic inputs. An increase in the frequency of presumed amacrine cell inputs is unexpected and may be a consequence of cannabinoid effects on inputs to these cells or may in fact represent a novel and unusual activation of responses by cannabinoid CB1 receptors.

A functional study by Cecyre et al. (2013) used electroretinogram (ERG) recordings in knockout mice for CB1 and CB2 receptors. Electroretinograms measure the population response of retinal cells in response to a light stimulus. A large number of cone photoreceptors respond in concert to a bright flash, followed by a slightly delayed response of second order cells, such as cone bipolar cells. A similar stimulus under dark-adapted conditions activates rod photoreceptors and the neurons downstream. This provides useful information about the light responses of these cells. Cecyre et al. reported that there was no difference in the ERG responses between wild type and CB1 knockout ERGs in response to a standard ERG light stimulus—a series of 1 ms flashes of progressively increasing intensity. A follow-on study from the same group reaffirmed a non-effect in ERGs for CB1 deletion, activation, or block (Cecyre et al., 2020). Given the functional photoreceptor data from retinal slices that show a pronounced effect both on the photoreceptor and bipolar cell responses (e.g., Fan and Yazulla, 2007), one possibility is that this difference is due to the brief nature of the light stimulus typically employed for ERGs. A 1 ms stimulus may not be sufficient to interrogate the role of a feedback signal such as that of the cannabinoid signaling system. Feedback loops require time for the signal to be received by photoreceptors, transmitted across the synapse, for changes in postsynaptic polarization, subsequent changes in calcium and endocannabinoid synthesis, for these lipid messengers to cross the synapse retrogradely and then act on the presynaptic photoreceptors. DSI/DSE, for example generally requires at least stimulation of 100 ms duration to induce and the following responses take place over the course of tens of seconds (e.g., Straiker and Mackie, 2005). However, as it stands there is a disconnect between the global electrical responses and those observed in retinal slice recordings for photoreceptors and bipolar cells.

## Retinal Ganglion Cells and Cannabinoid Signaling

In the inner retina, bipolar cells and amacrine cells serve to create a balance of excitatory and inhibitory inputs that ultimately determine the likelihood that a given retinal ganglion cell will fire an action potential. The likelihood of firing will therefore depend on the balance of excitatory and inhibitory inputs and the intrinsic membrane properties of the retinal ganglion cell. Several studies have looked at various aspects of this in whole-mount preparations, retinal slices, and acutely dissociated retinal ganglion cells.

Several studies made use of isolated cultured retinal ganglion cells. Lalonde et al. (2006) and Zhang et al. (2013) each examined cannabinoid responses in cultured rat retinal ganglion cells. As noted previously, CB1 receptors in neurons are, as a rule, expressed presynaptically. In the case of retinal ganglion cells, this would place the receptors outside the retina, in target regions of the visual circuitry. So, while interesting, the study of these cells may tell us more about likely function outside the retina. That said, Lalonde et al reported inhibition by the CB_1_ receptor agonist WIN55212 of high-voltage activated calcium currents in RGCs. Zhang et al. examined an outward potassium current, given the results of prior studies that found cannabinoid effects on potassium channels in goldfish (Fan and Yazulla, 2005). They found that WIN55212 suppressed the potassium current albeit at relatively high concentrations that were not blocked by CB_1_ or CB_2_ antagonists and the authors suggested that the effect occurred *via* a novel receptor target.

Other groups have made use of semi-intact preparations, either whole-mount or slices. Middleton and Protti (2011) showed using whole-mount retinas that spontaneous inputs onto retinal ganglion cells were inhibited by WIN55212. Both excitatory (glutamatergic) and inhibitory (GABAergic) inputs were diminished, though they saw a larger effect on the GABAergic currents in young mice, indicating that there is a variation with age and that the balance between excitatory and inhibitory inputs may change with time. Their results were consistent with a presynaptic expression of CB_1_ receptors onto the inputs. Jiang et al. (2013) recorded retinal ganglion cell firing properties using current-clamp recordings in retinal slices of rats (3–4 weeks of age) to examine whether the intrinsic firing and membrane properties of retinal ganglion cells were altered by cannabinoids. As noted above, CB_1_ receptors are typically located at the axon terminal, and so would have been cut away as a part of the slice preparation. Their non-effect is consistent with this, though they report that the action potential itself was altered though it is unclear to what extent the effect is CB1-dependent.

Wang et al. (2016) using retinal slices presented evidence that depolarization of retinal ganglion cells was able to suppress mIPSC inputs to these cells, potentially consistent with depolarization-induced suppression of inhibition (DSI). More recently, Middleton et al. (2019) reported that inputs to a specific population of ganglion cells—ON sustained—saw a CB1-dependent reduction in their spontaneous activity and altered spatial tuning.

**Outlook**: In reports of ganglion cell function, all cells see alterations of their inputs, suggesting a more global effect of cannabinoids. This may relate to a further caveat of any studies of the IPL in semi-intact or intact preparations, namely, the extent to which responses in cells of the inner nuclear layer and the ganglion cell layer are altered upstream by cannabinoid modulation at the OPL synapses. Because CB_1_ staining is observed at most or even all presynaptic terminals in the OPL, perfusion with an agonist may have unpredictable consequences for signaling downstream. And those consequences may depend on light levels prior to and during recordings. They may also depend on diurnal factors since cannabinoid levels have been shown to vary by time of day (Valenti et al., 2004), and cycling FAAH levels have been shown to underlie diurnal regulation of anterior eye function (Miller et al., 2016).

Another important question, the answer to which will impact cannabinoid signaling, is the extent to which cannabinoids spread beyond existing synapses. Wilson and Nicoll (2001) showed that cannabinoids can spread and act beyond a given synapse. In the hippocampus, this spread extended as far as 40 microns, enough spread to reach nearly 30 rod photoreceptors in either direction in the mouse (Carter-Dawson and LaVail, 1979). Until very recently, it has not been possible to directly observe the release of eCBs, and studies have been forced to rely on indirect means of detecting the consequences of cannabinoid signaling, but a new modified fluorescing CB_1_ receptor appears to serve as an endocannabinoid sensor and may at last offer insight into this question (Liput et al., 2019).

## How Does The Role of CB_1_ Compare in These Two Sensory Systems?

At first glance, the visual and olfactory systems would seem to have much in common. In both systems, an external signal must be perceived and converted into an electrochemical signal that can then be processed and interpreted by the downstream neuronal network. The nature of the signal to be interpreted differs fundamentally—the retina captures light, while the olfactory system binds specific volatile chemicals—but in both cases, a dedicated class of sensory neurons plays this role of detecting the stimulus and producing a signal that can be interpreted by a network of downstream neurons. And CB_1_ receptors are present at several points, including the early synapses, of each sensory system.

But there are also fundamental differences between these systems. Much of this derives from the objective of each sensory system. The chief goal of the olfactory system is to interrogate the volatile chemical environment with its uncountable number of potential chemicals and to derive from this a chemical profile: a specific, recognizable, identifiable scent. The olfactory system accomplishes this by evaluating the collective binding of a given volatile molecule to 500 or so different olfactory receptors (Buck and Axel, 1991; Gilad and Lancet, 2003; Niimura, 2009; Mainland et al., 2013; Hayden and Teeling, 2014). The olfactory system is additionally tasked with locating the direction of the source of the scent. And there is neuronal machinery in place to rapidly desensitize to a specific scent, presumably to allow detection of a sequential series of scents over the course of minutes. An intact olfactory system offers a tremendous benefit for food-seeking, predator aversion, and mating and some species such as rodents rely heavily on this sense to thrive. In contrast, the retina, as the vanguard of the visual system, uses a total of four receptor types with the objective of creating a multi-color, multidimensional representation of the outside world while adjusting for movement both of the observer and of objects in the outside world, and to changes in ambient light. To accomplish this requires highly complex circuitry, encompassing 60 or more neuronal types in the retina alone before preprocessed signals are sent to visual centers in the rest of the brain. Given the considerable differences between these systems, it is possible then that the roles of CB_1_ will be most comparable at early synapses.

Anatomically there appears to be a clear difference between the two sensory systems. In the retina, there is evidence for a retrograde feedback circuit onto cone photoreceptors and likely rods as well. In the olfactory bulb, this would be most comparable to the synapse between olfactory neurons and second order mitral cells in the glomeruli. Thus far, there is no compelling evidence for an active CB_1_ cannabinoid component circuit in the glomeruli themselves. Instead, the first defined cannabinoid circuit in the olfactory pathway is found on periglomerular interneurons synapsing onto the dendrites of mitral cells. The CB_1_ receptors are situated just outside the glomeruli. This circuit likely tempers the inhibitory inputs of these neurons onto mitral cells.

Another point of difference between the two systems has to do with the way they are integrated with the brain, more specifically with centrifugal inputs back to each sensory system. The olfactory bulb is more integrated with the rest of the brain, receiving centrifugal inputs from multiple brain regions (Kiselycznyk et al., 2006) and it has been shown that cannabinoids play a key role in regulating some of these inputs from the rest of the brain, with implications for food intake (Soria-Gómez et al., 2014). The mammalian retina, in contrast, is relatively more isolated from the brain and does not receive such centrifugal inputs, though other species such as birds do (Dillingham et al., 2013).

And lastly, the olfactory bulb includes a separate unit, the accessory olfactory bulb (Wackermannová et al., 2016; Smith and Bhatnagar, 2019). This component of the olfactory bulb receives qualitatively distinct inputs, partly related to pheromones. Though this system appears to have a relatively minor role in humans, in some species the accessory olfactory bulb is important to survival. The role of cannabinoids in this system is still unknown, but the system does not have a correlate in the visual sensory system.

The above-referenced studies on endocannabinoid signaling in the early stages of olfactory and visual signal processing indicate that despite the anatomical and physiological differences between these two sensory systems, CB_1_ and its associated enzymatic machinery are major players in the regulation of sensory input in both systems. It remains to be determined in future studies how this CB_1_-mediated regulation plays out at synaptic stages further along the visual and olfactory pathways.

**Outlook**: A full understanding of the parallels between cannabinoid signaling in the olfactory and visual pathways will require a greatly expanded understanding of the circuitry and function of these receptors in either system. With a few exceptions, the role of the cannabinoid signaling system in the visual pathway is still poorly understood. Though the circuitry at the first synapse is fairly well studied, we know little of the cannabinoid circuitry in the inner plexiform layer. The field also suffers from conflicting anatomical and functional studies, particularly between electrophysiological studies using retinal slices that report clear effects on photoreceptor signaling vs. studies that report no impact of CB1 on the ERG response. Functional studies of how cannabinoid receptors regulate the outputs of identified ganglion cell populations will be an important step toward this end. In both sensory systems, we have a limited understanding of the role of endogenous cannabinoids, which endocannabinoid plays a central role, as well as a detailed understanding of the enzymatic machinery that synthesizes and metabolizes these endocannabinoids. Newer tools such as an endocannabinoid sensor may shed some much-needed light on this subject in both sensory systems.

## Author Contributions

TH and AS designed the article and wrote the article. All authors contributed to the article and approved the submitted version.

## Conflict of Interest

The authors declare that the research was conducted in the absence of any commercial or financial relationships that could be construed as a potential conflict of interest.
